# Supplementary material for: Comparative multiomics analysis of cell physiological state after culture in a basket bioreactor
Source: Sci Rep. 2022 Nov 23;12:20161. doi: 10.1038/s41598-022-24687-4 (PMC9686226; doi:10.1038/s41598-022-24687-4)
Supplement: Supplementary file 1 — Supplementary Information 1. [file 41598_2022_24687_MOESM1_ESM.zip › raw data/Transcriptomic raw data/4.Quant/2.Correlation/PCA2D.pdf]

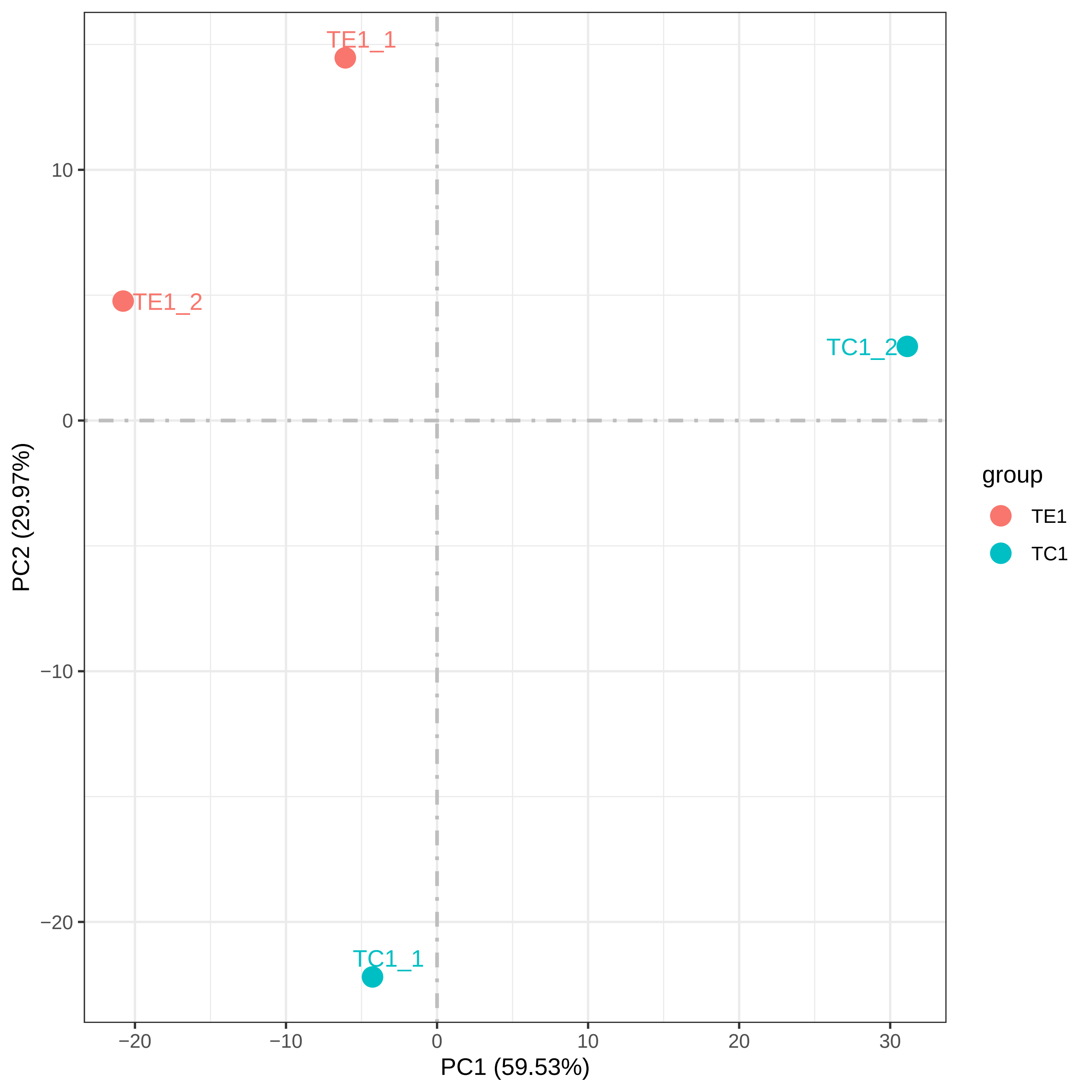
TE1_1
TE1_2
TC1_2
TC1_1
10
0
−10
−20
−20
−10
0
10
20
30
PC1 (59.53%)
PC2 (29.97%)
group
TE1
TC1
